# Multiple Compounds Secreted by *Pseudomonas aeruginosa* Increase the Tolerance of *Staphylococcus aureus* to the Antimicrobial Metals Copper and Silver

**DOI:** 10.1128/mSystems.00746-20

**Published:** 2020-09-08

**Authors:** Nadia K. Monych, Raymond J. Turner

**Affiliations:** a Department of Biological Sciences, University of Calgary, Calgary, Alberta, Canada; Lebanese American University

**Keywords:** *Pseudomonas aeruginosa*, *Staphylococcus aureus*, antimicrobial, bacterial interactions, copper, metal resistance, metal tolerance, polymicrobial, silver

## Abstract

Metal-based antimicrobials have been used for thousands of years to treat and prevent bacterial infections. Currently, both silver and copper are used in health care and industry to prevent and treat the spread of harmful bacteria. However, like most antimicrobial agents, their efficacy against polymicrobial infections has not been fully elucidated. Coinfection with Pseudomonas aeruginosa and Staphylococcus aureus and the resulting interactions have been implicated in higher virulence, antibiotic resistance, and increased chronic infections. Here, the influence of secreted compounds from P. aeruginosa on metal antimicrobial tolerance in S. aureus was examined. This study determined that multiple compounds from P. aeruginosa increase the tolerance of S. aureus to copper and/or silver when cultured in simulated wound fluid. The presence of these secreted compounds from P. aeruginosa during exposure of S. aureus to copper or silver increased the MIC from 500 μM to 2,000 μM for copper and 16 to 63 μM for silver. The contribution of specific compounds to S. aureus tolerance was determined using gene deletion and disruption mutants, and metabolite analysis. Compounds identified as potential contributors were then individually added to S. aureus during metal exposure. Copper tolerance in S. aureus was found to be increased by amino acids and dihydroaeruginoate (Dha) secreted by P. aeruginosa. The silver tolerance provided to S. aureus was influenced only by two amino acids, serine and threonine, as well as the *Pseudomonas* quinolone signal (PQS) molecules from P. aeruginosa.

**IMPORTANCE** Alternative antimicrobials, such as metals, are one of the methods currently used to help mitigate antibiotic resistance. Metal-based antimicrobials such as copper and silver are used currently both to prevent and to treat infections. Although the efficacy of these antimicrobials has been determined in single-species culture, bacteria rarely exist in a single-species group in the environment. Both Pseudomonas aeruginosa and Staphylococcus aureus are often found associated with each other in severe chronic infections displaying increased virulence and antibiotic tolerance. In this study, we determined that multiple compounds secreted by P. aeruginosa are able to increase the tolerance of S. aureus to both copper and silver. This work demonstrates the expansive chemical communication occurring in polymicrobial infections between bacteria.

## Abstract (precis)

Alternative antimicrobials, such as metals, are one of the methods currently used to help mitigate antibiotic resistance. Metal-based antimicrobials such as copper and silver are used currently both to prevent and to treat infections. Although the efficacy of these antimicrobials has been determined in single-species culture, bacteria rarely exist in a single-species group in the environment. Both Pseudomonas aeruginosa and Staphylococcus aureus are often found associated with each other in severe chronic infections displaying increased virulence and antibiotic tolerance. In this study, we determined that multiple compounds secreted by P. aeruginosa are able to increase the tolerance of S. aureus to both copper and silver. This work demonstrates the expansive chemical communication occurring in polymicrobial infections between bacteria.

## INTRODUCTION

The presence of multidrug-resistant bacteria in health care and industry has seen a drastic increase over the past 20 years (1–4). Due to the ever-evolving state of microorganisms, research has been focused on overcoming antibiotic resistance through the development and use of alternative antimicrobial compounds, including metals (5–8). The use of metals as antimicrobials can be dated back to ancient civilizations including the Egyptians and Phoenicians (9, 10). These civilizations would drop coins into their water vessels or coat them to keep the water “fresh” during transport (9, 10). Currently, metals such as copper and silver are used in hospitals for indwelling medical devices, copper-coated surfaces, silver eyedrops, and bandages (11–13). Water treatment facilities also use copper-silver ionization systems to prevent bacterial contamination (14).

Two pathogens of increasing concern to health organizations around the world are Pseudomonas aeruginosa and Staphylococcus aureus (15, 16). These organisms are associated with each other in severe chronic infections such as cystic fibrosis (17–19). Silver and copper are often used to assist wound healing and for the prevention and treatment of chronic infections (20–24). The study of polymicrobial infections and interactions has begun to develop only over the past 10 years (25–29). During this period, several reviews and articles have been published examining both antagonistic and synergistic interactions between P. aeruginosa and S. aureus (19, 30–34). Some of these studies observed differences in antibiotic tolerance of either P. aeruginosa or S. aureus with secreted products from the other organism (32–36). Compounds from P. aeruginosa including siderophores, 2-heptyl-4-hydroxyquinoline *n*-oxide (HQNO), pyocyanin, and acylated homoserine lactones (AHLs) were all implicated in changes to antibiotic tolerance of S. aureus (32, 33). The influence of these compounds was also dependent on the strain, antibiotic, and growth conditions used (32–35).

Similarly to investigations into antibiotic efficacy, testing of different metal antimicrobial formulations is normally performed on a single species. However, our group has observed that similarly to antibiotics, metal susceptibility differs between single species and cocultured bacteria (37, 38). Particularly, when P. aeruginosa and S. aureus were cultured together, a higher MIC was observed for AgNO_3_ than when either was cultured individually (37, 38). This indicates these organisms were more tolerant to silver when grown together.

The purpose of this work was to explore the influence of secreted molecules from one strain on individual metal susceptibility of the other strain. Here, we focus on compounds secreted by P. aeruginosa which influence the tolerance of S. aureus toward silver and copper metal-based antimicrobials. Extracts, and fractions thereof, from spent medium of P. aeruginosa grown in simulated wound fluid (SWF) were evaluated, and compounds that contributed to increased metal tolerance were identified. The findings presented here show a system of multiple biomolecules involved that demonstrates that this resistance effect is multifactorial, where different compounds differentially influence the tolerance of different metals (Ag versus Cu).

## RESULTS

### Pseudomonas aeruginosa PAO1 enhances tolerance of S. aureus ATCC 25923 to copper and silver.

The spent medium, containing secreted compounds from each organism during growth in simulated wound fluid (SWF) medium, was collected and added to the opposing organism exposed to AgNO_3_ (see Fig. S1 in the supplemental material). This experiment revealed that a compound(s) contained within P. aeruginosa spent medium (PaS) was responsible for enhancing silver tolerance. The enhanced tolerance was also specific to SWF, as the use of other media to prepare and test the spent medium resulted in either no change or reduced AgNO_3_ tolerance (Fig. S2). The ability of PaS to provide tolerance to S. aureus was determined for multiple metals, metalloids, antibiotics, and antiseptics (Table S1). Although PaS either reduced or had no impact on S. aureus tolerance for many of these antimicrobials, both copper and silver tolerance was enhanced. The addition of PaS to S. aureus was able to provide a 4-fold increase to the MIC of copper and silver (Fig. S3). Initial inhibition of S. aureus alone exposed to AgNO_3_ and CuSO_4_ was 16 and 500 μM, respectively (Fig. S3). For S. aureus in the presence of PaS, the MIC increased to 63 μM for AgNO_3_ and 2,000 μM for CuSO_4_, denoted by the inflection point that occurs at these concentrations (Fig. S3). It should be noted that although tolerance of S. aureus alone to CuSO_4_ appears to increase slightly after 1,000 μM, this is due to slight absorbance of copper in the medium at these high concentrations.

10.1128/mSystems.00746-20.1 FIG S1 S. aureus (a) or P. aeruginosa (b) exposed to 0 to 500 μM AgNO_3_ with (red square) and without (black circle) addition of spent medium from either P. aeruginosa (PaS) (a) or S. aureus (SaS) (b). Statistical differences between either organism alone and with spent medium were determined using Welch’s *t* test for three biological trials with three technical replicates each. There was no significant difference between P. aeruginosa with or without SaS for any concentration of AgNO_3_. The *P* values for S. aureus alone or with PaS for each concentration of AgNO_3_ are represented by * (<0.05), ** (<0.01), and *** (<0.001). Download FIG S1, TIF file, 1.3 MB. Copyright © 2020 Monych and Turner. 2020 Monych and Turner This content is distributed under the terms of the Creative Commons Attribution 4.0 International license.

10.1128/mSystems.00746-20.2 FIG S2 Optical density at 600 nm of S. aureus after exposure to multiple concentrations of AgNO_3_ in different media with and without spent medium prepared in the same medium. Spent medium from P. aeruginosa was prepared after culturing in either CSWF (chemically simulated wound fluid) (Pa^CSWF^), LB (Pa^LB^), or M9Cas (Pa^M9Cas^). S. aureus was then exposed to different concentrations of AgNO_3_ from 0 to 500 μM for 24 h at 37°C with 150-rpm shaking in either CSWF (a), LB (b), or M9Cas (c) with or without addition of the corresponding spent medium. Values are the average from three biological trials with three technical replicates each. Statistical differences between S. aureus alone and with spent medium for each AgNO_3_ concentration were determined using a Welch *t* test. Resulting *P* values are represented as * (<0.05), ** (<0.01), and *** (<0.001). Download FIG S2, TIF file, 2.3 MB. Copyright © 2020 Monych and Turner. 2020 Monych and Turner This content is distributed under the terms of the Creative Commons Attribution 4.0 International license.

10.1128/mSystems.00746-20.3 FIG S3 S. aureus exposed to 0 to 4,000 μM CuSO_4_ (a) or 0 to 500 μM AgNO_3_ (b) with added P. aeruginosa spent medium (PaS) (red square) or without it (black circle). The optical density at 600 nm of S. aureus after 24 h at 37°C with 150-rpm shaking was recorded for each condition. Individual normalized tolerance values are displayed with the average and standard deviation from three biological trials with three technical replicates each. A one-way ANOVA was used to determine significant differences between S. aureus alone and with PaS for each metal concentration. Resulting *P* values are displayed as ** (<0.01) and *** (<0.001). Download FIG S3, TIF file, 1.2 MB. Copyright © 2020 Monych and Turner. 2020 Monych and Turner This content is distributed under the terms of the Creative Commons Attribution 4.0 International license.

10.1128/mSystems.00746-20.9 TABLE S1 Initial MICs of different metal(loid)s, antibiotics, and antiseptics for S. aureus alone or with P. aeruginosa spent medium (PaS). Download Table S1, DOCX file, 0.1 MB. Copyright © 2020 Monych and Turner. 2020 Monych and Turner This content is distributed under the terms of the Creative Commons Attribution 4.0 International license.

### Multiple compounds from P. aeruginosa are able to enhance copper and/or silver tolerance in S. aureus.

Further experiments were performed to characterize and identify the compound(s) able to enhance tolerance of S. aureus to copper and silver. Preliminary characterization experiments were used to determine the heat tolerance, hydrophobicity, and approximate size of the tolerance-providing compound (Fig. S4). The spent medium was either treated with heat at 95°C for 30 min or separated with a 2:1 chloroform-methanol extraction. The spent medium was also filtered through 50-, 30-, 10-, or 3-kDa-molecular-weight-cutoff filters either prior to or after these treatments. Based on these experiments and subsequent separation on a μ reverse-phase chromatography (μRPC) C_2_-C_18_ column, we observed multiple fractions that differed in their ability to provide copper and/or silver tolerance (Fig. S4 and S5).

10.1128/mSystems.00746-20.4 FIG S4 S. aureus exposed to either 600 μM CuSO_4_ (a) or 50 μM AgNO_3_ (b) with or without (SWF) spent medium from P. aeruginosa after heat treatment (PaH95) or chloroform-methanol extraction (PaMC and PaAq) as well as after subsequent molecular weight filtration through 50-, 30-, 10-, or 3-kDa filters for heat-treated (Pa50H95, Pa30H95, and Pa10H95) and aqueous (Pa50Aq, Pa30Aq, Pa10Aq, and Pa3Aq) PaS. Optical density at 600 nm (OD_600_) of the exposed S. aureus after 24 h at 37°C and 150-rpm shaking was recorded and normalized to represent tolerance changes as individual values with the average and standard deviation for three biological trials with three technical replicates each. A Welch *t* test was used to determine any significant difference between S. aureus alone (SWF) and with additional spent medium. The *P* values are represented as * (<0.05), ** (<0.01), and *** (<0.001). Download FIG S4, TIF file, 1.1 MB. Copyright © 2020 Monych and Turner. 2020 Monych and Turner This content is distributed under the terms of the Creative Commons Attribution 4.0 International license.

10.1128/mSystems.00746-20.5 FIG S5 Representative data of the separation of PaAq on a μRPC-C_2_-C_18_ column with a gradient indicated with a solid line from eluent A (water with 0.065% TFA) to eluent B (acetonitrile with 0.05% TFA). Flowthrough from the column was collected as 0.5-ml fractions for 6 ml followed by a gradient from 0 to 100% eluent B where 2-ml fractions were collected for 8 ml as indicated by the dotted lines on the spectra. Elution of compounds was followed at 3 different wavelengths: 280-nm blue, 210-nm red, and 360-nm green lines (a). Resulting fractions were combined and dried under cold N_2_ gas and then suspended in 100 μl PBS and sterilized. Optical density readings at 600 nm of S. aureus exposed to 50 μM AgNO_3_ alone (SWF) or with the indicated fractions after 24 h at 37°C with 150-rpm shaking were normalized and plotted with the average and standard deviation for three biological trials with three technical replicates each (b). Statistical significance of S. aureus exposure in medium alone (SWF) and with the fractions was calculated with a Welch *t* test with *P* values represented as * (<0.05), ** (<0.01), and *** (<0.001). Download FIG S5, TIF file, 1.3 MB. Copyright © 2020 Monych and Turner. 2020 Monych and Turner This content is distributed under the terms of the Creative Commons Attribution 4.0 International license.

**FIG 1:**
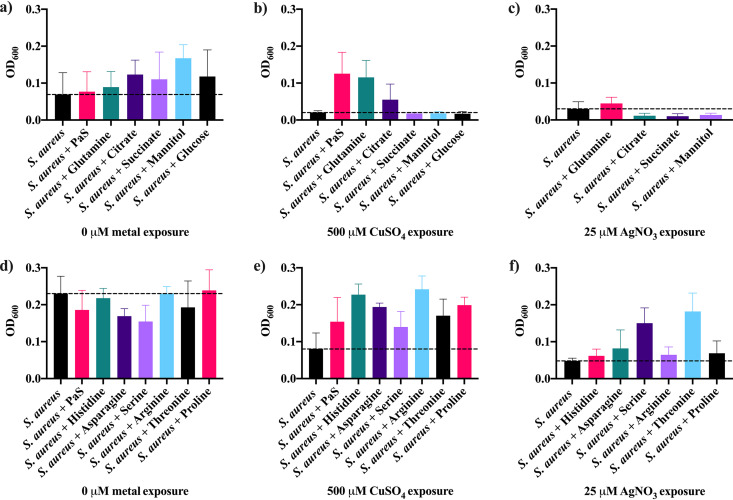
Optical density at 600 nm (OD_600_) of S. aureus alone (a and d) or after exposure to either 500 μM CuSO_4_ (b and e) or 25 μM AgNO_3_ (c and f) with and without additional metabolites for 24 h at 37°C with 150-rpm shaking. Either 10 mM glutamine, citrate, succinate, mannitol, or glucose (a, b, and c) or histidine, asparagine, serine, arginine, threonine, or proline (d, e, and f) or 16% PaS was added to S. aureus. The average and standard deviation for three biological trials with three technical replicates each were plotted. The dashed line represents the tolerance of S. aureus alone with no additives.

The compounds which provided both copper and silver tolerance that were hydrophilic, were below 3 kDa, and eluted as a passthrough peak (fraction 3) using a μRPC-C_2_-C_18_ column were sent for metabolic analysis. A list of compounds present in this fraction was obtained (data not shown), and the most abundant of these (>5 × 10^7^ relative abundance) were individually added to S. aureus exposed to copper or silver (Fig. 1). Glucose and citrate were also included as they may affect either S. aureus fitness or metal binding. The fitness of S. aureus unexposed to any metal was enhanced only with mannitol and citrate (Fig. 1a) though copper and silver tolerance was not impacted by these metabolites (Fig. 1b and c). All of the amino acids tested were able to enhance copper tolerance of S. aureus (Fig. 1e). However, none of the amino acids used increased the fitness of S. aureus without metal exposure (Fig. 1d). Silver tolerance of S. aureus was increased only with the addition of serine or threonine (Fig. 1f).

### Siderophores and quorum sensing molecules from P. aeruginosa influence S. aureus copper and silver tolerance.

While not identified during metabolic analysis (a ramification of liquid chromatography-tandem mass spectrometry [LC-MS/MS] conditions), other compounds which could impact S. aureus tolerance include siderophores, pili and flagellar proteins, pyocyanin, hydrogen cyanide, or quorum sensing systems. Using purified pyocyanin, we found that it is not involved in our system (Fig. S6). While cyanide was able to increase S. aureus silver and copper tolerance, this was observed only at a concentration above what would be found in the spent medium under our conditions (39) (Fig. S7).

10.1128/mSystems.00746-20.6 FIG S6 Representative data of the crude purification and susceptibility testing of added pyocyanin in the bioassay. Pyocyanin was purified from P. aeruginosa spent medium (PaS) extracted twice with chloroform, dried under cold N_2_ gas, and suspended in PBS (Pa^2Cl^). The aqueous fraction from the extraction was also collected, dried under cold N_2_ gas, and suspended in PBS (Pa^aq^). A 100-μl aliquot of Pa^2Cl^ was loaded onto a μRPC C_2_-C_18_ column. The column was washed with 2 column volumes of solvent A (0.065% TFA in MilliQ H_2_O) followed by a gradient from 0 to 100% 0.05% TFA in acetonitrile for 9 column volumes where 1-ml fractions were collected at a flow rate of 0.5 ml/min. (a) Absorbance was recorded at 210, 280, and 400 nm, and the fraction containing a sharp peak at 400 nm was collected, dried, and suspended in 100 μl PBS (PaF10). (b and c) The optical density at 600 nm of S. aureus after exposure for 24 h at 37°C with 150-rpm shaking to either 600 μM CuSO_4_ (b) or 40 μMAgNO_3_ (c) with and without (SWF) addition of PaS, Pa^aq^, Pa^2Cl^, and PaF10 was recorded for two biological replicates with three technical replicates each. Statistical differences between S. aureus exposed to metal alone (SWF) and that with additional spent medium were determined using a Welch *t* test with *P* values represented as * (<0.05), ** (<0.01), and *** (<0.001). Download FIG S6, TIF file, 1.1 MB. Copyright © 2020 Monych and Turner. 2020 Monych and Turner This content is distributed under the terms of the Creative Commons Attribution 4.0 International license.

10.1128/mSystems.00746-20.7 FIG S7 S. aureus exposed to either 40 μM AgNO_3_ (a) or 500 μM CuSO_4_ (b) with and without either 10 μM or 500 μM KCN. Optical density at 600 nm (OD_600_) of the exposed S. aureus after 24 h at 37°C and 150-rpm shaking was recorded and normalized to represent tolerance changes as individual values with the average and standard deviation for three biological trials with three technical replicates each. A one-way ANOVA was used to determine any significant difference between S. aureus alone (SWF) and with KCN. The *P* values are represented as * (<0.05), ** (<0.01), and *** (<0.001). Download FIG S7, TIF file, 1.0 MB. Copyright © 2020 Monych and Turner. 2020 Monych and Turner This content is distributed under the terms of the Creative Commons Attribution 4.0 International license.

We performed further experiments utilizing full gene deletion mutants and transposon disruption mutants for genes involved in siderophore synthesis (*pchE*, *pchF*, and *pvdD*), quorum sensing (*lasI*, *lasR*, *rhlI*, *rhlR*, *pqsA*, *pqsH*, *pqsL*, and *mvfR*), and pili (*pilA*) and flagellar (*fliC*) proteins from P. aeruginosa PAO1. The spent medium for each of these mutants was then collected in the same way as for wild-type P. aeruginosa and added to S. aureus to examine changes to either copper or silver tolerance. If the product of the deleted or disrupted gene was involved in enhancing either copper or silver tolerance, the spent medium from that mutant would no longer provide tolerance to S. aureus. Opposing this, if the product of the gene had no influence on copper or silver tolerance the provided enhancement would be the same for the mutant as wild-type spent medium.

**FIG 2:**
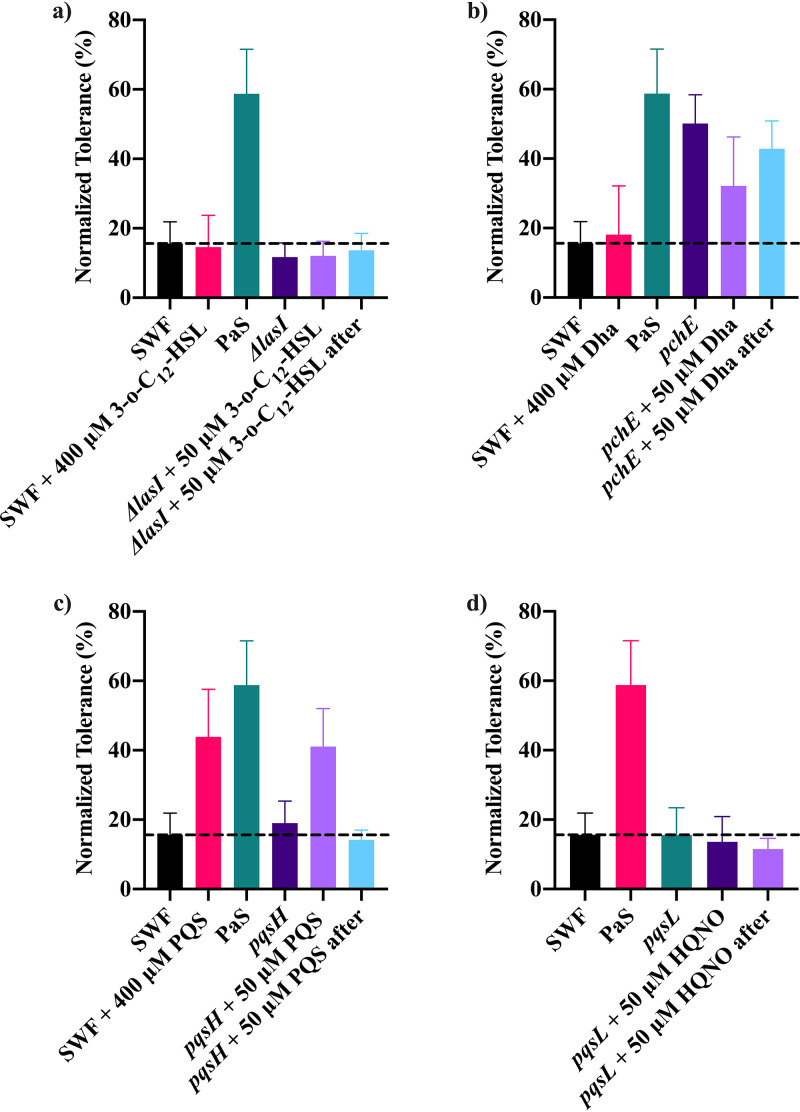
The normalized tolerance of S. aureus to 40 μM AgNO_3_ was determined using a bioassay with and without the addition of spent medium from wild-type P. aeruginosa PAO1 (PaS) or gene deletion or disruption mutants for *lasI* (a), *pchE* (b), *pqsH* (c), or *pqsL* (d). Spent medium was also prepared with the addition of 50 μM 3-*o*-C_12_-HSL to *lasI*, 50 μM Dha to *pchE*, 50 μM PQS to *pqsH*, and 50 μM HQNO to *pqsL* during and after culturing. A higher concentration (400 μM) of each of the compounds was also added to S. aureus exposed to silver alone during the bioassay. The average and standard deviation from three biological trials with three technical replicates each are plotted. The dashed line represents the copper tolerance of S. aureus alone (SWF).

The silver tolerance provided by P. aeruginosa spent medium was unaffected by the absence of *fliC*, *pchF*, or *mvfR* (Fig. S8). The absence of either *pilA* or *pvdD* in P. aeruginosa prevented its spent medium from increasing tolerance of S. aureus to silver (Fig. S8). Spent medium from the *pqsA* transposon (Tn) mutant provided only a partial tolerance phenotype to S. aureus (Fig. S8). Finally, the disruption of *pchE* in P. aeruginosa spent medium also had no influence on the conferred silver tolerance (Fig. 2).

10.1128/mSystems.00746-20.8 FIG S8 S. aureus exposed to either 25 μM AgNO_3_ (a and b) or 500 μM CuSO_4_ (c and d) with and without (SWF) spent medium from wild-type P. aeruginosa (PaS) or different gene deletion and transposon mutants. Spent medium was prepared from gene deletion mutants of P. aeruginosa PAO1 for *pilA*, *fliC*, *lasR*, *lasI*, *rhlR*, and *rhlI* as well as transposon mutants for *pchF* and *pvdD* (a and c) as well as *pqsA* and *mvfR* (b and d). Optical density at 600 nm of S. aureus was recorded after 24 h at 37°C with 150-rpm shaking. The normalized tolerance determined from the optical density readings is indicated with the average and standard deviation after a minimum of three biological trials with three technical replicates each. The statistical significance was determined using a Welch *t* test comparing both between S. aureus alone and with spent medium, with *P* values represented by * (<0.05), ** (<0.01), and *** (<0.001), as well as between S. aureus with PaS and each mutant spent medium indicated with lines and stars. Download FIG S8, TIF file, 1.7 MB. Copyright © 2020 Monych and Turner. 2020 Monych and Turner This content is distributed under the terms of the Creative Commons Attribution 4.0 International license.

Both the *las* and *rhl* quorum sensing systems appeared to be involved in the spent media’s ability to confer silver tolerance. Spent media prepared from gene deletion mutants for *lasR*, *lasI*, *rhlR*, and *rhlI* were all unable to provide a significant enhancement to silver tolerance in S. aureus (Fig. S8). To examine the influence of one of these systems more closely, the signal molecule 3-*o*-C_12_-HSL (homoserine lactone) used in the *las* system was added to S. aureus alone and during the preparation of both mutant and wild-type spent media (Fig. 2a). The exogenous addition of 3-*o*-C_12_-HSL to S. aureus during silver exposure did not increase silver tolerance (Fig. 2a). The use of *lasI* spent medium prepared with addition of 3-*o*-C_12_-HSL during the growth phase and after collection also had no enhancement to silver tolerance (Fig. 2a).

While the transcriptional regulator MvfR is not involved in the provided silver tolerance, other parts of the *pqs* system are. Disruption of *pqsH* or *pqsL* reduced the ability of P. aeruginosa spent medium to increase S. aureus silver tolerance (Fig. 2c and d). The protein products of *pqsH* and *pqsL*, 2-heptyl-3-hydroxy-4(1H)-quinolone synthase and monooxygenase, are responsible for the final step in synthesis of *Pseudomonas* quinolone signal (PQS) and 2-heptyl-4-hydroxyquinoline *n*-oxide (HQNO), respectively (40, 41). The effect of both HQNO and PQS individually on S. aureus silver tolerance and their ability to recover their respective mutant’s ability to increase silver tolerance were also examined. When HQNO was added to the *pqsL* Tn mutant either during or after growth, the resulting spent medium was unable to increase silver tolerance (Fig. 2d). Spent medium was also prepared from the *pqsH* Tn mutant with addition of PQS during and after culturing. The addition of 50 μM PQS to *pqsH* spent medium during its growth was able to recover the ability to increase S. aureus silver tolerance (Fig. 2c). Although the addition of a lower concentration (50 μM) of PQS to the *pqsH* spent medium after growth was not enough to increase silver tolerance in S. aureus, 400 μM PQS alone was able to protect S. aureus from silver toxicity (Fig. 2c).

**FIG 3:**
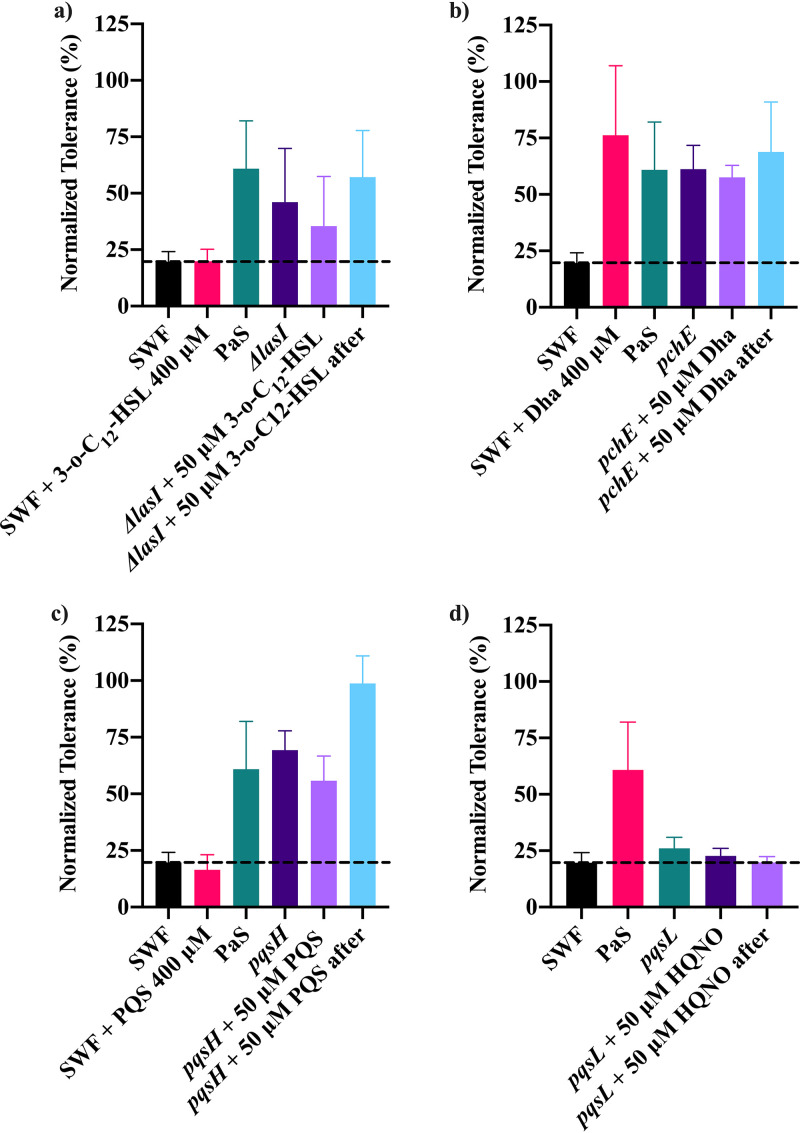
The normalized tolerance of S. aureus to 500 μM CuSO_4_ was determined using a bioassay with and without the addition of spent medium from wild-type P. aeruginosa PAO1 (PaS) or gene deletion or disruption mutants for *lasI* (a), *pchE* (b), *pqsH* (c), or *pqsL* (d). Spent medium was also prepared with the addition of 50 μM 3-*o*-C_12_-HSL to *lasI*, 50 μM Dha to *pchE*, 50 μM PQS to *pqsH*, and 50 μM HQNO to *pqsL* during and after culturing. A higher concentration (400 μM) of each of the compounds was also added to S. aureus exposed to copper alone during the bioassay. The average and standard deviation from three biological trials with three technical replicates each are plotted. The dashed line represents the copper tolerance of S. aureus alone (SWF).

Unlike for silver tolerance, the provided copper tolerance was mostly unaffected by quorum sensing systems. The mutant spent media from deletion or disruption mutants for *lasR*, *rhlR*, *rhlI*, *mvfR*, *pqsA*, and *pqsH* were still able to provide copper tolerance to S. aureus (Fig. S8). The absence of *pilA*, *fliC*, *pchF*, or *pvdD* from P. aeruginosa also had no significant impact on the spent medium’s ability to confer copper tolerance (Fig. S8). The use of spent medium from the *lasI* gene deletion mutant was unable to increase copper tolerance compared to S. aureus alone, but this was not significantly lower than the tolerance provided by wild-type PaS (Fig. 3a). The addition of exogenous 3-*o*-C_12_-HSL directly to the bioassay or during growth of Δ*lasI* spent medium also did not impact copper tolerance (Fig. 3a). Though the addition of 3-*o*-C_12_-HSL to Δ*lasI* spent medium after its preparation was able to increase copper tolerance, this was very minor.

The only gene which appeared to have a significant influence on copper tolerance was *pqsL*, as its removal resulted in a significant reduction in the conferred copper tolerance (Fig. 3d). The role of *pqsL* in conferring copper tolerance is similar to what was observed for silver tolerance. Neither the addition of HQNO to *pqsL* Tn mutant spent medium during nor addition after growth was able to recover the spent medium’s ability to increase copper tolerance (Fig. 3d).

While the primary siderophore in P. aeruginosa, pyoverdine, does not influence copper tolerance, pyochelin does, particularly the precursor to pyochelin, dihydroaeruginoic acid (Dha). The *pchE* Tn mutant spent medium was still able to confer copper tolerance to S. aureus, but exogenous Dha also increased S. aureus copper tolerance (Fig. 3b).

## DISCUSSION

The impact of interspecies interactions on antimicrobial tolerance presents a significant issue during treatment of chronic infections. By understanding the factors which increase or decrease the metal tolerance during interspecies interactions, preventative measures may be taken. Previous reports identified an increased tolerance to AgNO_3_ during coculture of P. aeruginosa and S. aureus compared to their individual cultures (37, 38). However, a mechanism responsible for this observation was not proposed. The findings from the current study indicate that multiple compounds secreted by P. aeruginosa can increase S. aureus tolerance to AgNO_3_ and CuSO_4_. In contrast to this, compounds secreted by S. aureus have no significant impact on tolerance of P. aeruginosa to AgNO_3_ (see Fig. S1 in the supplemental material).

Although previous reports identified a link between pyocyanin and silver tolerance provided by P. aeruginosa, this was not the case in our system using SWF as the culture medium (35, 42). Other researchers have also reported a link between hydrogen cyanide and interactions between P. aeruginosa and S. aureus (33). Though the presence of hydrogen cyanide did increase both silver and copper tolerance, the concentration needed is above what would be secreted by P. aeruginosa (39).

The compounds present in P. aeruginosa spent medium that impact AgNO_3_ tolerance differ from those influencing CuSO_4_ tolerance. In both cases, binding interactions which reduce the bioavailability of the metal in the medium influence the observed tolerance. However, different binding coordination chemistry and affinities are involved for each metal. The conferred silver tolerance was directly linked to serine, threonine, and PQS. The ability of serine and threonine to increase silver tolerance was largely unexpected, as they are not normally associated with silver atom binding sites (43). The addition of serine or threonine did not increase S. aureus growth without metal exposure, signifying that increased fitness is unlikely to be the cause of the increased silver tolerance. Instead, this observation could be due to the enzymes used to metabolize serine and threonine in S. aureus. These amino acids are generally the first to be metabolized during amino acid catabolism due to their easy conversion to pyruvate (44). Part of this conversion involves an l-serine dehydratase containing an iron-sulfur cluster (44). Targeting of iron-sulfur clusters by silver is one of the proposed mechanisms of toxicity (45). Thus, Ag attack on the dehydratase would lead to an excess of serine or threonine able to bind up the silver, protecting S. aureus from other detrimental effects.

Quorum sensing in P. aeruginosa is also involved as the spent media from gene deletion mutants for *lasR*, *lasI*, *rhlR*, and *rhlI* did not enhance silver tolerance in S. aureus. The *lasR* and *lasI* genes code for the transcriptional activator protein LasR, the regulator for the *las* system, and an acyl-homoserine-lactone synthase which produces the inducer molecule *N*-(3-oxododecanoyl)-l-homoserine lactone (3-*o*-C_12_-HSL) (46). Similarly, *rhlR*’s protein product is the regulator for the *rhl* system, regulatory protein RhlR, and *rhlI*’s protein product is another acyl-homoserine-lactone synthase which produces *N*-butanoyl-l-homoserine lactone (BHL) and *N*-hexanoyl-l-homoserine lactone (HHL), both autoinducers for the *rhl* system (47, 48). The *las* and *rhl* systems regulate a large amount of P. aeruginosa’s genome and are highly interconnected (49). The absence of any these genes would cause a shift in the compounds produced, any of which could explain the reduction in provided tolerance.

The ability of PaS to provide silver tolerance to S. aureus is also associated with production of the *Pseudomonas* quinolone signal (PQS) but does not require the functional regulator, encoded by *mvfR*, in P. aeruginosa. Both spent medium from mutant *pqsH* P. aeruginosa grown in the presence of a lower concentration (50 μM) of PQS and that from PQS alone at a higher concentration (400 μM) were able to increase silver tolerance. This indicates that PQS can directly increase S. aureus silver tolerance at a high-enough concentration or signal within active P. aeruginosa causing production of another compound(s) which increases silver tolerance in S. aureus (49–53). PQS by itself is involved in both outer membrane vesicle generation and siderophore production in P. aeruginosa (53). An increase in either of these in the medium would reduce the bioavailability of silver through its binding, thus providing an increase to S. aureus tolerance. Additionally, PQS has been positively correlated with biofilm production in S. aureus (54). These outcomes of PQS-altered physiology increase tolerance to silver for S. aureus.

The ability of spent medium from P. aeruginosa to confer copper tolerance to S. aureus is directly linked to the presence of amino acids and Dha. Exogenous Dha provided an increase to copper tolerance in S. aureus. However, the disruption of *pchE*, coding for dihydroaeruginoic acid (Dha) synthetase responsible for synthesizing Dha from salicylic acid, does not change tolerance (55). This suggests that although Dha helps S. aureus copper tolerance, it is not a necessary component of P. aeruginosa spent medium to provide copper tolerance. Binding interactions of the various secreted compounds reducing copper bioavailability are the best explanation for the copper tolerance provided by amino acids or Dha.

The conferred silver and copper tolerances were also reduced when either *lasI* or *pqsL* was absent, but they could not be restored by addition of either 3-*o*-C_12_-HSL or HQNO. Since the protein products of *lasI* and *pqsL* are responsible for the last step of 3-*o*-C_12_-HSL and HQNO synthesis, respectively, if either 3-*o*-C_12_-HSL or HQNO was directly involved in copper or silver tolerance, their exogenous addition would be expected to increase S. aureus tolerance. This was not the case, implying a more indirect mechanism of involvement for *lasI* and *pqsL*. When either *lasI* or *pqsL* is disrupted, there is likely a change to the yet-unidentified compounds secreted by P. aeruginosa. This change in physiology then alters the ability of spent medium from either the *lasI* or *pqsL* mutants to increase silver or copper tolerance.

Challenges in this study arise from the variability of our data between experiments. This is a product of the challenge recovery assessment of the remaining fitness distribution of the cells in a culture after antimicrobial metal load. Differences in fitness possibilities of the community depend on the medium augmentation as well as other factors that would influence culture growth and density, including, but not limited to, number of phase variants, persister cells, or mutations that likely arise. Additionally, there can be remarkable differences in medium batches for antimicrobial testing (this is why clinical tests often use comparative breakpoints). This is increased when fetal bovine serum (FBS) is used as this medium component of SWF is undefined, leading to compositional differences between batches. These differences can result in slight changes to S. aureus physiological fitness and associated growth. The subtle differences in medium and cell composition can also lead to different metal ion speciation states (how the metal ion is coordinated and to what), altering the effective bioavailability and associated toxicity. There would also be slight genetic/phenotypic variation in the colonies picked between biological replicates. This is in part alleviated by comparing an average of technical replicates within a single biological replicate, yet there is still considerable variance at times between the magnitudes of the observed phenotype between replicates. These issues led to different metal tolerance levels during our study, and thus, the challenge concentrations used were altered in some experiments to ensure that inhibition occurred. Particularly, silver seemed to be more affected by these variations. All these considerations in combination with the cross signaling discovered in this study demonstrate how complex a mixed-species wound environment system is and the diversity of responses to antimicrobial treatment that is observed.

**FIG 4:**
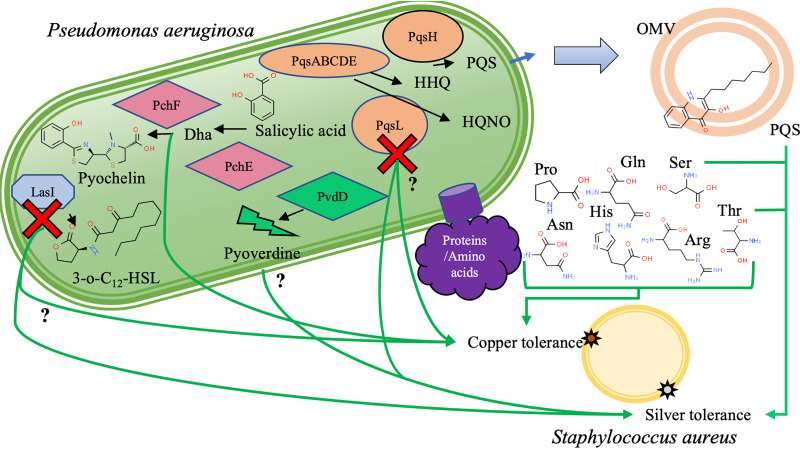
Summary of the compounds or genes involved in providing either copper or silver tolerance to S. aureus. Green arrows represent compounds influencing copper or silver tolerance, and the question mark indicates a minor impact that was not confirmed by addition of the individual compound. The red “x” represents disruption of either *pqsL*, coding for a probable flavin adenine dinucleotide (FAD)-dependent monooxygenase which produces HQNO, or *lasI*, coding for an acyl-homoserine-lactone synthase which produces 3-*o*-C_12_-HSL. While this disruption reduces the provided tolerance from P. aeruginosa spent medium, loss of HQNO or 3-*o*-C_12_-HSL is not the cause. Other compounds involved with either copper or silver tolerance enhancement include dihydroaeruginoic acid (Dha), the *Pseudomonas* quinolone signal (PQS) likely secreted through outer membrane vesicles (OMV), serine, threonine, histidine, arginine, asparagine, proline, and glutamine.

Overall, S. aureus is protected from both copper and silver toxicity in the presence of spent medium from P. aeruginosa when grown specifically in simulated wound fluid medium. The secretion of PQS or compounds controlled directly by PQS, as well as serine and threonine, all provides silver tolerance to S. aureus. These compounds likely act in combination to reduce the bioavailability of silver in the medium through binding (PQS) as well as affecting S. aureus’ metabolism and physiology (PQS, serine, and threonine) to create a more tolerant phenotype. A similar mechanism occurs when protecting from copper; however, different compounds are involved. Compounds which bind copper to reduce its bioavailability include amino acids and Dha. While neither HQNO nor 3-*o*-C_12_-HSL directly impacts silver or copper tolerance, the absence or dysfunction of the proteins which synthesize them leads to a loss of the P. aeruginosa causing spent media’s ability to confer tolerance (summarized in Fig. 4). Our results suggest that further cell components are involved in this metal resistance communication and that even with a binary bacterial system there is remarkable complexity.

## MATERIALS AND METHODS

### Bacterial strains and culture maintenance.

The strains used in this study include Pseudomonas aeruginosa PAO1 and Staphylococcus aureus ATCC 25923. Gene deletion mutants in P. aeruginosa PAO1 including *lasR*, *lasI*, *rhlR*, *rhlI*, *pilA*, and *fliC* were gifts from J. Harrison, and transposon mutants for *pchE*, *pchF*, *pvdD*, *pqsA*, *pqsH*, *pqsL*, and *mvfR* were obtained from I. Lewis, both at the University of Calgary. All strains were prepared by streaking from a −80°C glycerol stock onto a Luria-Bertani (LB) agar plate and growing overnight at 37°C. A single colony from this primary plate was then streaked onto a secondary LB agar plate and grown overnight at 37°C. Colonies from the secondary plate were then suspended in saline (0.9% NaCl in distilled-deionized H_2_O [ddH_2_O]) to match a 1.0 McFarland standard, creating a standardized inoculum. This suspension was diluted into simulated wound fluid (SWF; 50% fetal bovine serum, 50% peptone water [0.1 g/liter peptone in 0.85% NaCl]) for culturing.

### Metal solutions.

Metal stock solutions were prepared in distilled and deionized H_2_O (ddH_2_O), sterilized through an 0.2-μm filter, and stored at room temperature, except for AgNO_3_, which was stored covered at 4°C. CuSO_4_ was prepared at 1 M, AgNO_3_ at 50 mM, and NiSO_4_ and AlSO_4_ at 100 mM. A working solution of metal was prepared by diluting the stock solution in sterile ddH_2_O before addition to the medium to prevent precipitation.

### Spent medium preparation.

Spent media, or the sterile secreted compounds, from either P. aeruginosa or S. aureus were prepared identically. The standardized inoculum for either P. aeruginosa or S. aureus was diluted 30-fold in a 96-well plate containing SWF. The culture was then grown for 24 h with 150-rpm shaking at 37°C until an optical density at 600 nm (OD_600_) of ∼1.0 was reached. The culture was collected in microcentrifuge tubes and centrifuged at 8,000 × *g* for 10 min. Spent medium was collected by filter sterilizing the resulting supernatant through an 0.2-μm syringe filter. Spent medium obtained from P. aeruginosa culture is referred to as PaS while spent medium from S. aureus culture is referred to as SaS. Further separation of spent medium was performed by filtration through molecular-weight-cutoff filters of 50, 30, 10, and 3 kDa in size. The temperature stability was also determined by heating at 95°C for 30 min. The hydrophobic and hydrophilic compounds were extracted from the spent medium using a 2:1 chloroform-methanol extraction.

### Antimicrobial susceptibility determination.

The standardized inoculum from either P. aeruginosa or S. aureus was diluted 300-fold into a 96-well plate containing 2-fold serial dilutions of either CuSO_4_ (63 to 4,000 μM), NiSO_4_ (63 to 4,000 μM), AgNO_3_ (16 to 500 μM), AlSO_4_ (500 to 8,000 μM), K_2_TeO_3_ (130 to 8,000 μM), Na_2_SeO_3_ (130 to 8,000 μM), NaAsO_2_ (130 to 8,000 μM), nalidixic acid (6.25 to 200 μg/ml), tetracycline (6.25 to 200 μg/ml), benzalkonium chloride (0.78 to 50 μg/ml), or H_2_O_2_ (1.6 to 8,000 μM). To determine the influence of spent medium on antimicrobial susceptibility, 16% of either PaS or SaS was also added to the previous plate. The 96-well plate containing the standardized inoculum, metal challenge, and spent medium was grown for 24 h at 37°C with 150-rpm shaking. The optical density at 600 nm was then recorded with a PerkinElmer 2030 Victor X4 microplate reader. The MIC was determined by the initial concentration in which there was a significant reduction in visible cell growth as read by optical density at 600 nm (OD_600_). Range finding experiments explored other concentrations of spent medium, time endpoints, and medium choice for this assay; the described assay method provided the clearest phenotype. The above protocol is based on a previous protocol with minor alterations for accommodating spent medium (56).

### Normalization.

The OD_600_ readings during some trials of the antimicrobial susceptibility determinations were lower for unexposed S. aureus alone than S. aureus with PaS. Due to this variance in optical density, a normalization was performed on the optical density readings to correct for the difference. The OD_600_ values for S. aureus both alone and with PaS were normalized between 0 and 100%. The optical density of both S. aureus alone and with PaS without metal exposure was set to 100% and an optical density reading of 0% to 0%. The optical density of S. aureus alone or with PaS during metal exposure was then set within the appropriate range whether PaS was present or not. This normalization allows for accurate comparison of susceptibility changes for S. aureus alone or with PaS.

### Optical density bioassay (BA^OD^).

The increased tolerance of S. aureus to AgNO_3_ and CuSO_4_ with spent medium from P. aeruginosa was tested using an optical density bioassay (BA^OD^). A 96-well plate was prepared with wells containing either SWF alone, SWF with 500 or 600 μM CuSO_4_, SWF with 25, 40, or 50 μM AgNO_3_, SWF with 500 or 600 μM CuSO_4_ and 16% spent medium, or SWF with 25, 40, or 50 μM AgNO_3_ and 16% spent media. Multiple spent medium types were tested in each bioassay always at the same concentration (16%). The plate was then inoculated with a 300-fold dilution of the standardized inoculum of S. aureus and incubated at 37°C for 24 h with 150-rpm shaking.

The optical density at 600 nm was then determined with a PerkinElmer 2030 Victor X4 microplate reader. The optical density was normalized and plotted as normalized tolerance (%) where the optical density of S. aureus without metal exposure was set to 100% and an optical density reading of 0% to 0%.

### Fraction separation using reverse-phase chromatography (RPC) and fraction testing with the bioassay.

A μRPC C_2_-C_18_ ST 4.6/100 column (Pharmacia Biotech) was used during all RPC separation and stored in 70% methanol until use. Into the column, 100 μl of either PaS, Pa10Aq, or Pa3Aq was injected, and the column was then equilibrated with eluent A (Milli-Q H_2_O with 0.065% trifluoroacetic acid [TFA]) for 1 column volume, followed by a gradient from 0 to 100% eluent B (acetonitrile with 0.05% TFA) for 10 column volumes with an 0.5-ml/minute flow rate, collecting 0.5-ml fractions during flowthrough and 2-ml fractions during the gradient. The resulting fractions were dried under cold N_2_ gas and suspended in 100 μl phosphate-buffered saline (PBS) and then autoclaved prior to addition to the bioassay at a 16% concentration.

### LC-HR MS/MS analysis.

Three biological replicates were prepared from fraction 3 of Pa3Aq separated using the previous RPC protocol. However, after the fraction was dried it was suspended in 50% methanol. The sample was processed at the Calgary Metabolomics Research Facility (CMRF) at the University of Calgary for metabolite separation and analysis using high-resolution liquid chromatography (LC-HR) MS. A Vanquish ultrahigh-performance LC (UHPLC) system (Thermo-Fisher) was used for sample injection and separation on a Syncronis HILIC column (Thermo-Fisher).The following conditions were used for separation: solvent A (20 mM ammonium formate, pH 3.0, in MS-grade H_2_O), solvent B (0.1% formic acid in MS-grade acetonitrile), and a gradient of 2 min at 100% B, 5 min to 80% B, 3 min to 5% B, 2 min at 5% B, and 1 min to 100% B at a flow rate of 0.6 ml/minute. A Q Exactive HF hybrid quadrupole-Orbitrap mass spectrometer (Thermo-Fisher) was then used with the following parameters: HESI source parameters were sheath gas flow rate 25, auxiliary gas flow rate 10, sweep gas flow rate 2, spray voltage 2.5 kV, capillary temperature 275°C, S-lens radio frequency level 60, and auxiliary gas heater temperature 325°C. MS scan parameters were runtime 15 min, negative polarity, full MS scan type, 240,000 resolution, automatic gain control (AGC) target of 3e6, interaction time maximum of 200 ms, and scan range between 50 and 750 *m/z*. The resulting files were analyzed using the MAVEN software program to detect compounds comparing to a list of known standards that had been previously separated using the same instrument (57, 58).

### Metabolite addition for bioassay.

The effect of individual metabolites on S. aureus tolerance to metals was determined following a similar protocol as the previous bioassay. However, instead of addition of spent medium, a 10 mM concentration of either glutamine, citrate, succinate, mannitol, glucose, histidine, asparagine, serine, arginine, threonine, or proline was added to the 96-well plate described in the bioassay. With these data, no normalization was performed to display the effect of metabolites on S. aureus fitness without metal exposure.

### Gene deletion and disruption mutant spent medium preparation and bioassay.

Stock solutions of *N*-(3-oxododecanoyl)-l-homoserine lactone (3-*o*-C_12_-HSL), dihydroaeruginoic acid (Dha), 2-heptyl-4-hydroxyquinoline *n*-oxide (HQNO), and *Pseudomonas* quinolone signal (PQS) were prepared at 10 mM in dimethyl sulfoxide (DMSO). Spent medium for Δ*lasI* was prepared as described previously but with 50 μM 3-*o*-C_12_-HSL added to the culture before and after growth. Spent media from the *pchE*, *pqsL*, and *pqsH* Tn mutants were prepared similarly to Δ*lasI* with 3-*o*-C_12_-HSL but using their corresponding compound (Dha to *pchE*, HQNO to *pqsL*, and PQS to *pqsH*). This spent medium as well as each compound alone at a 400 μM concentration was added to S. aureus exposed to either 40 μM AgNO_3_ or 500 μM CuSO_4_ in the bioassay described previously.

### S. aureus cyanide exposure bioassay.

A stock solution of 1 M KCN was prepared in Milli-Q H_2_O and filter sterilized with an 0.2-μm filter syringe system. The stock KCN was subsequently diluted to a working concentration in SWF prior to use in the previously described bioassay. Three 96-well plates were prepared containing either no KCN, 10 μM KCN, or 500 μM KCN. Each of the plates also contained wells with SWF without KCN or metal exposure, 40 μM AgNO_3_ with appropriate KCN addition, and 500 μM CuSO_4_ with appropriate KCN addition. S. aureus was then inoculated into each plate, cultured, and processed as described in the bioassay.

### Statistics.

Statistical differences were determined using a Welch *t* test, one-way analysis of variance (ANOVA) or a two-way ANOVA with Dunnett’s multiple comparisons using the GraphPad Prism version 8.2 for Mac, GraphPad Software, La Jolla, CA, USA.
