# Supplementary figures and images for: Multiple Compounds Secreted by Pseudomonas aeruginosa Increase the Tolerance of Staphylococcus aureus to the Antimicrobial Metals Copper and Silver
Source: mSystems. 2020 Sep 8;5(5):e00746-20. doi: 10.1128/mSystems.00746-20 (PMC7483513; doi:10.1128/mSystems.00746-20)

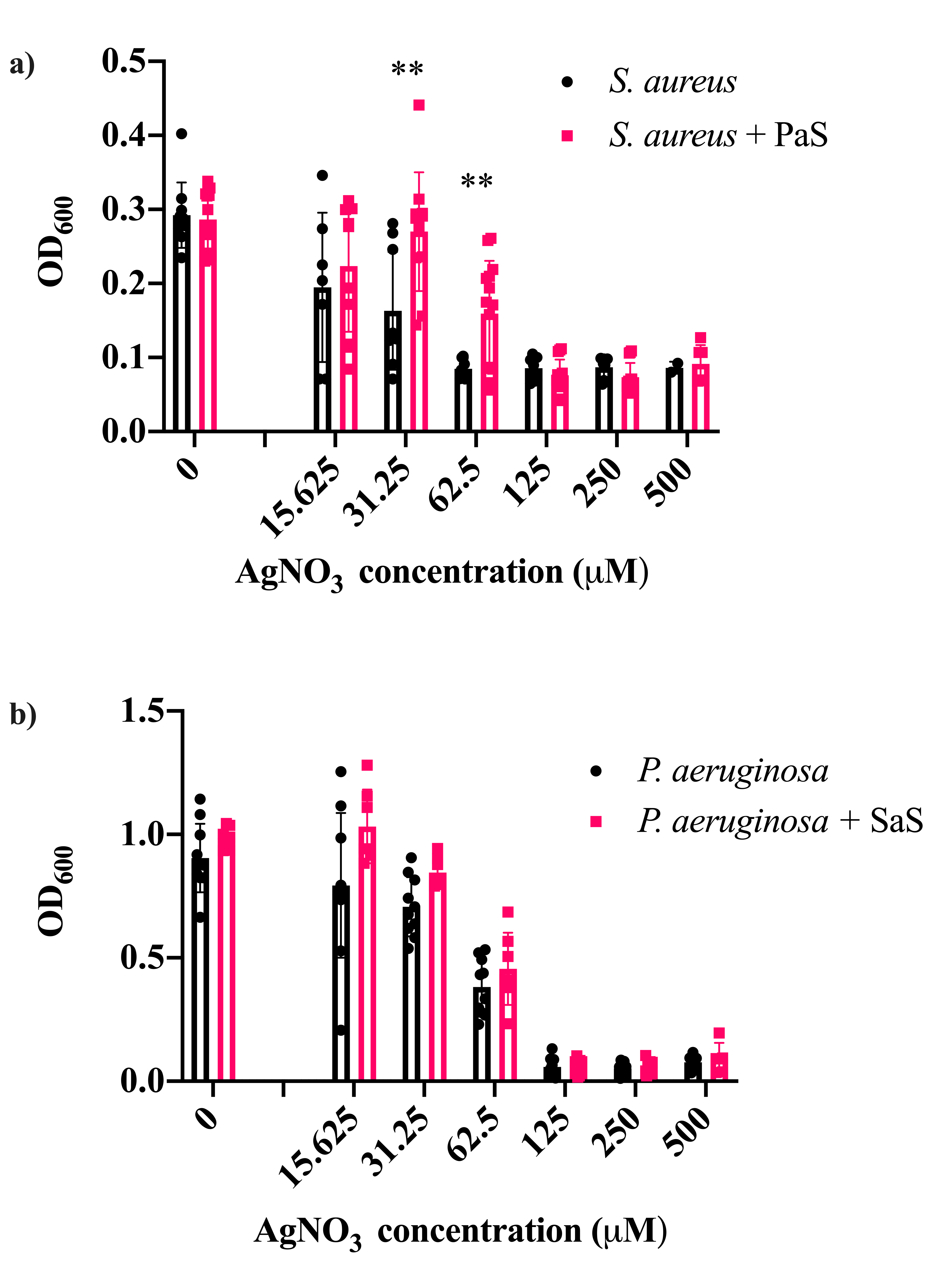

Supplement: FIG S1 [file mSystems.00746-20-sf001.tif]

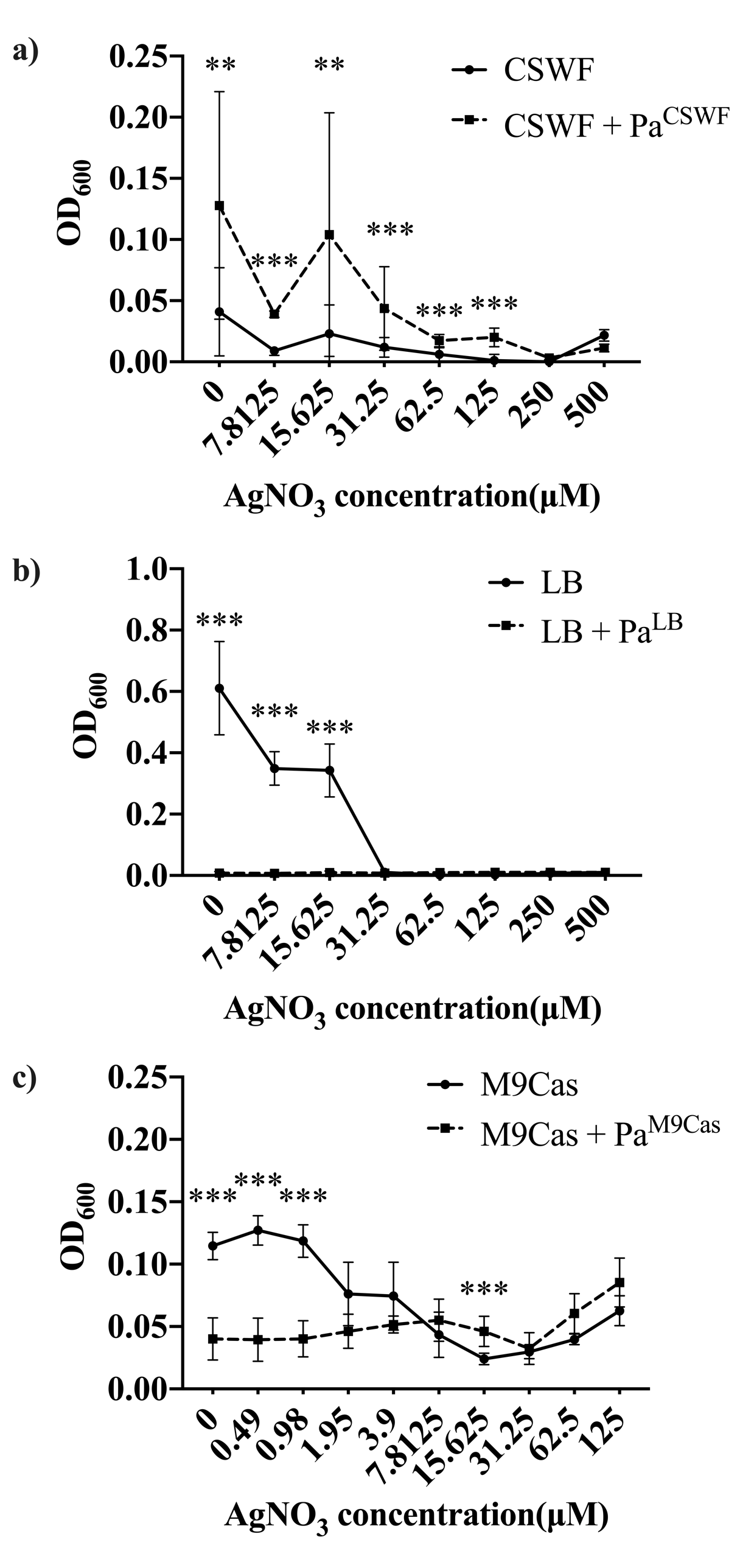

Supplement: FIG S2 [file mSystems.00746-20-sf002.tif]

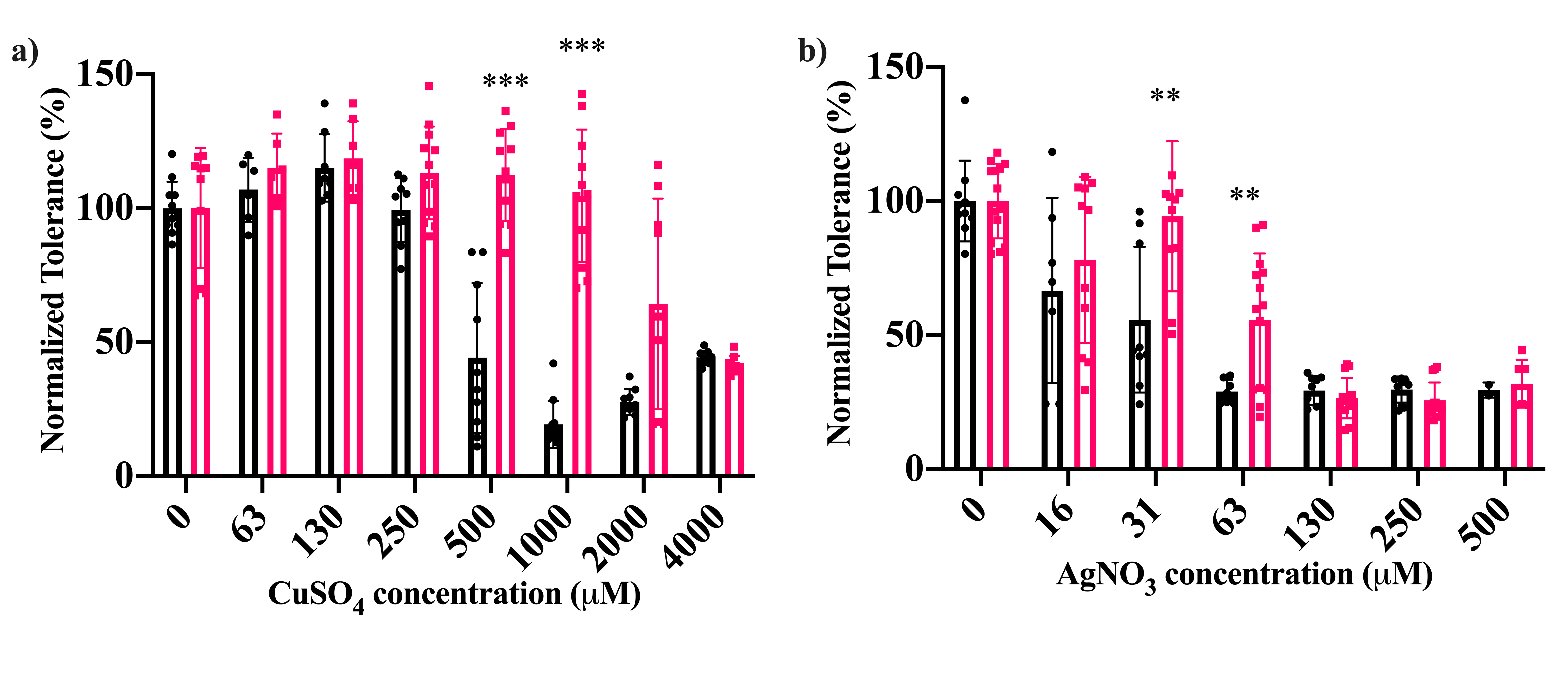

Supplement: FIG S3 [file mSystems.00746-20-sf003.tif]

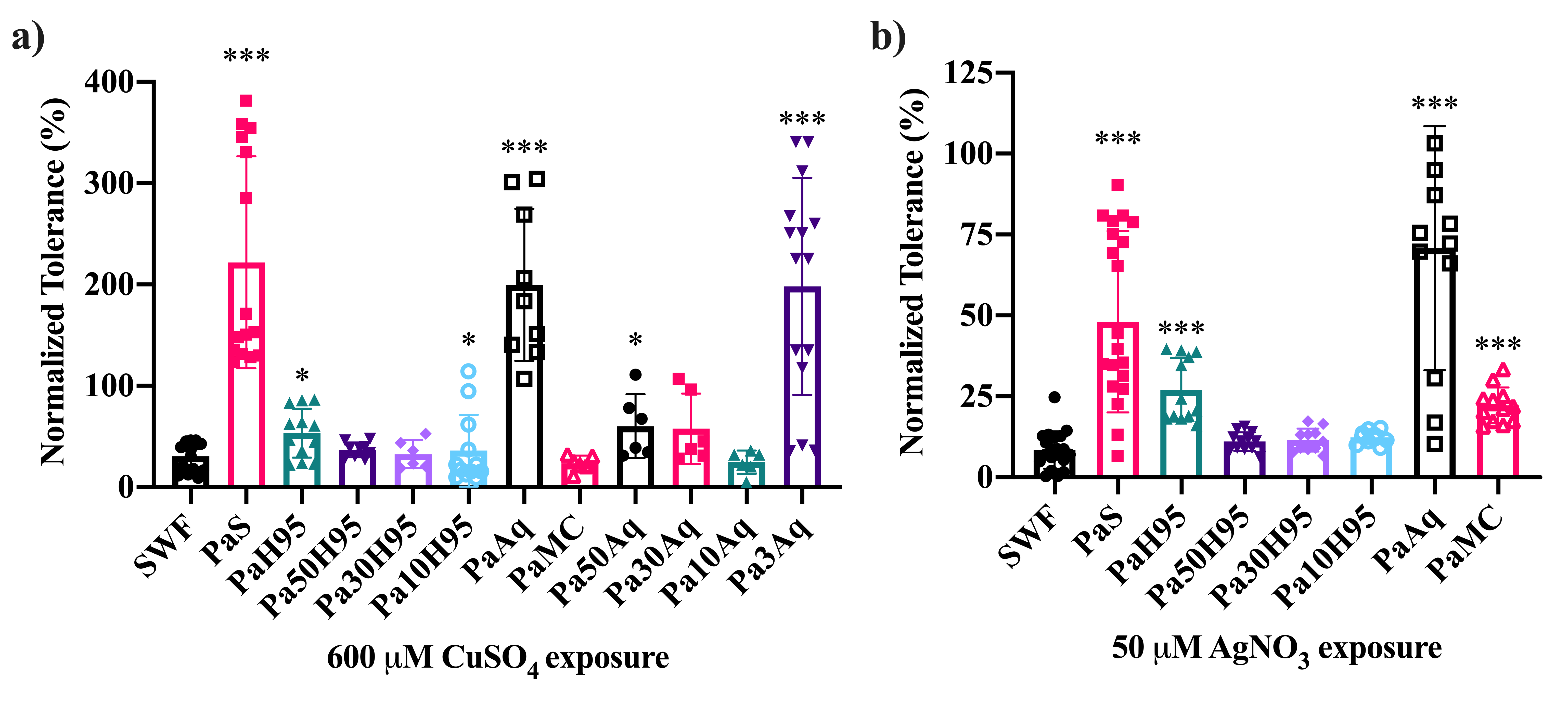

Supplement: FIG S4 [file mSystems.00746-20-sf004.tif]

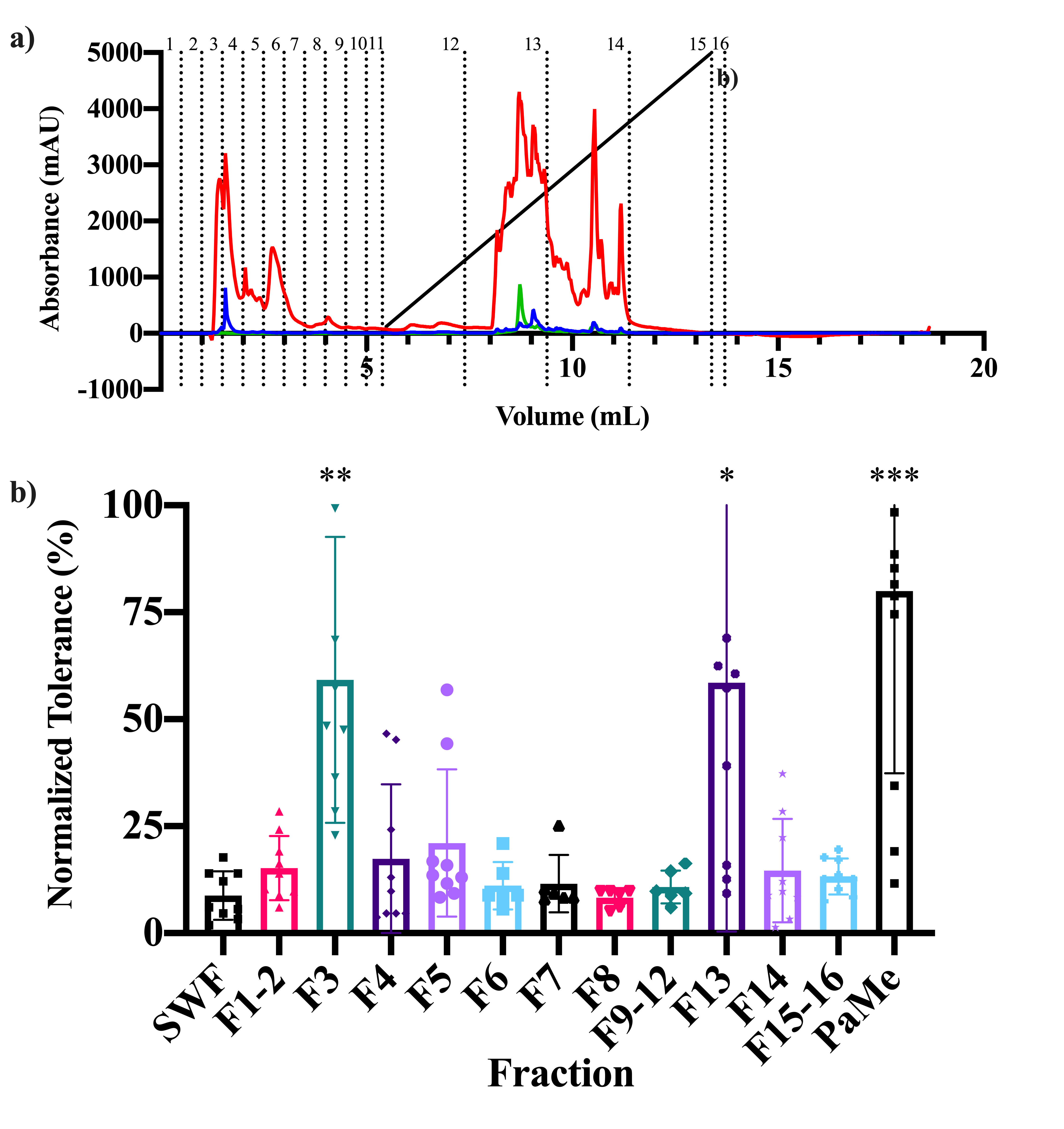

Supplement: FIG S5 [file mSystems.00746-20-sf005.tif]

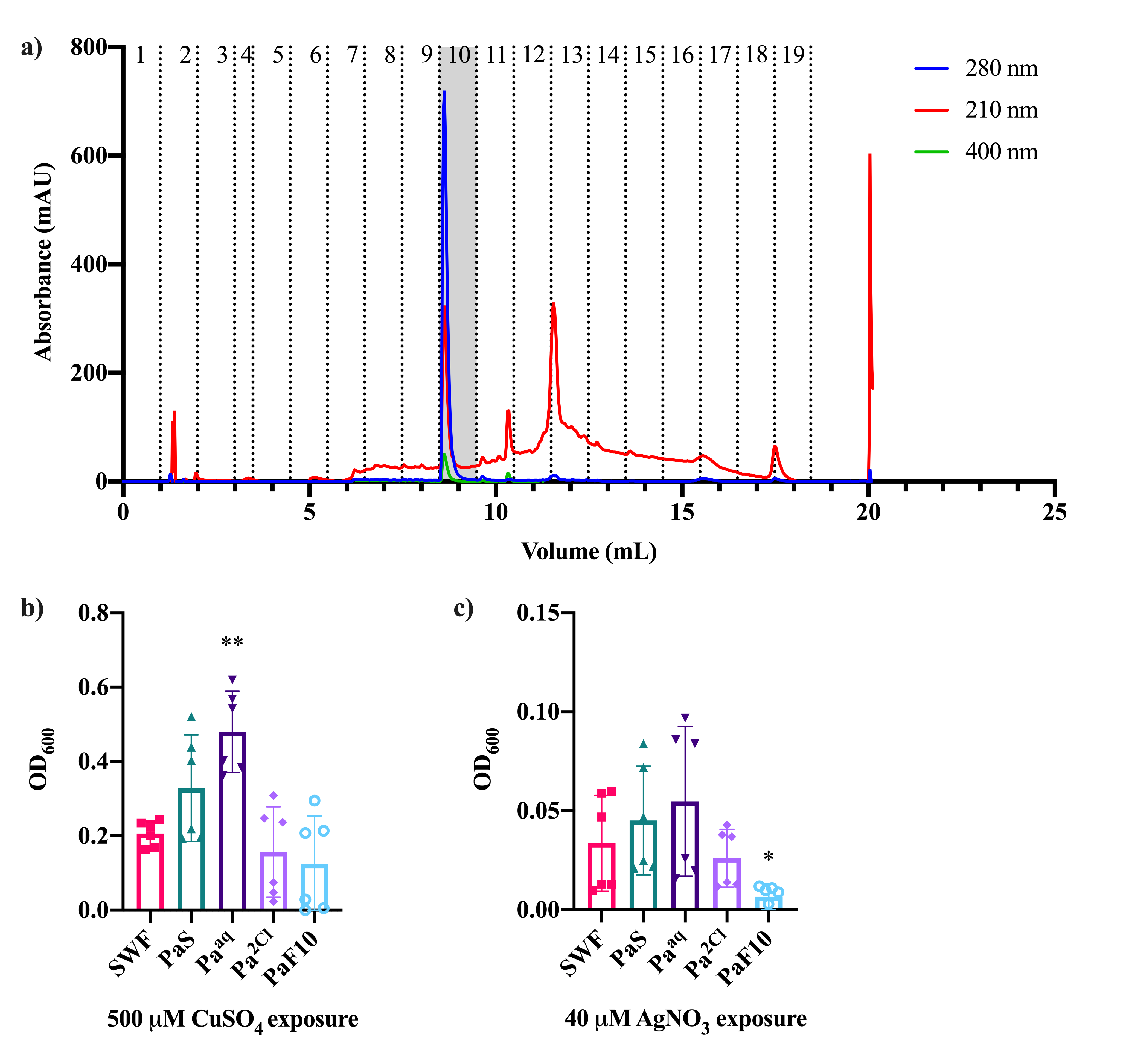

Supplement: FIG S6 [file mSystems.00746-20-sf006.tif]

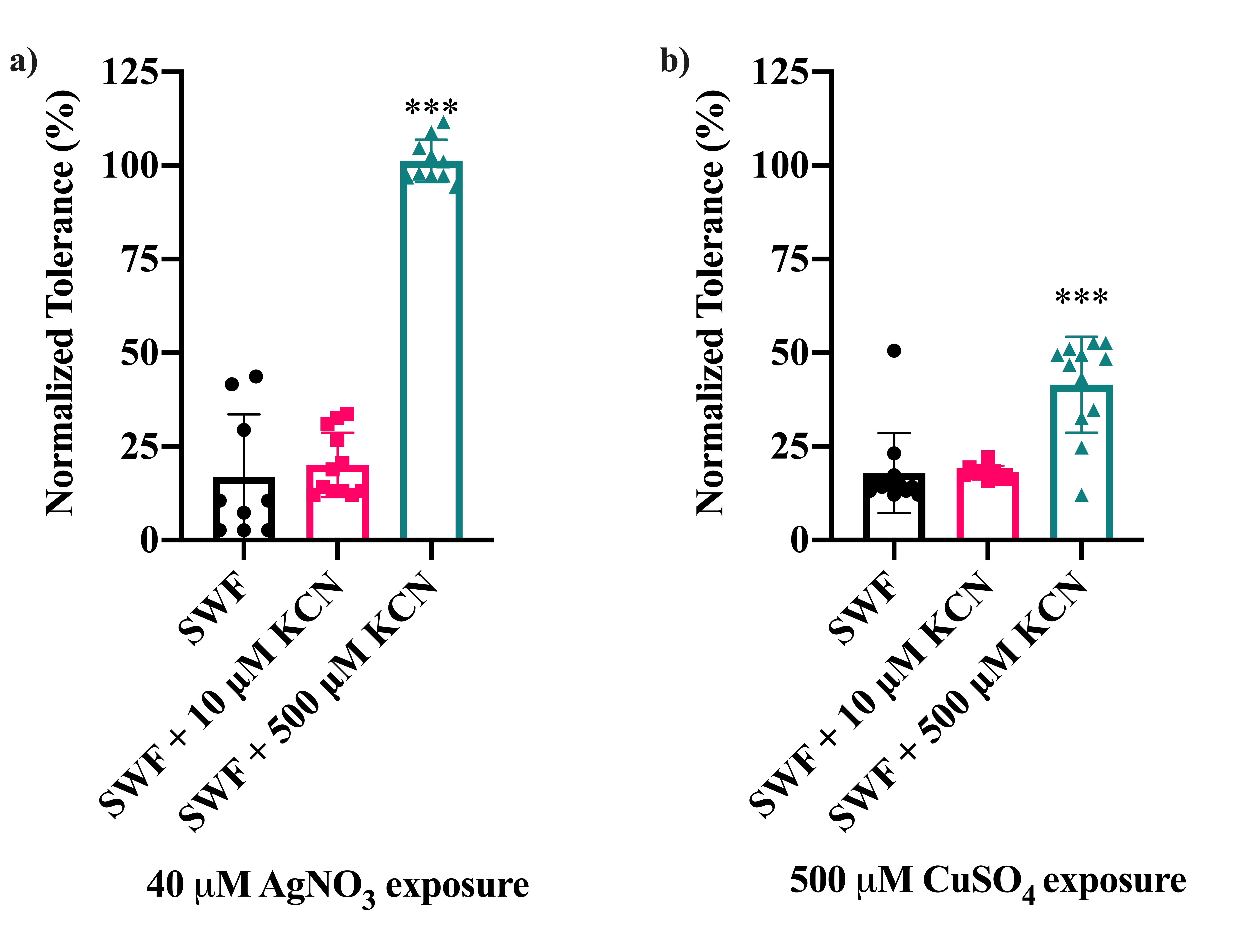

Supplement: FIG S7 [file mSystems.00746-20-sf007.tif]

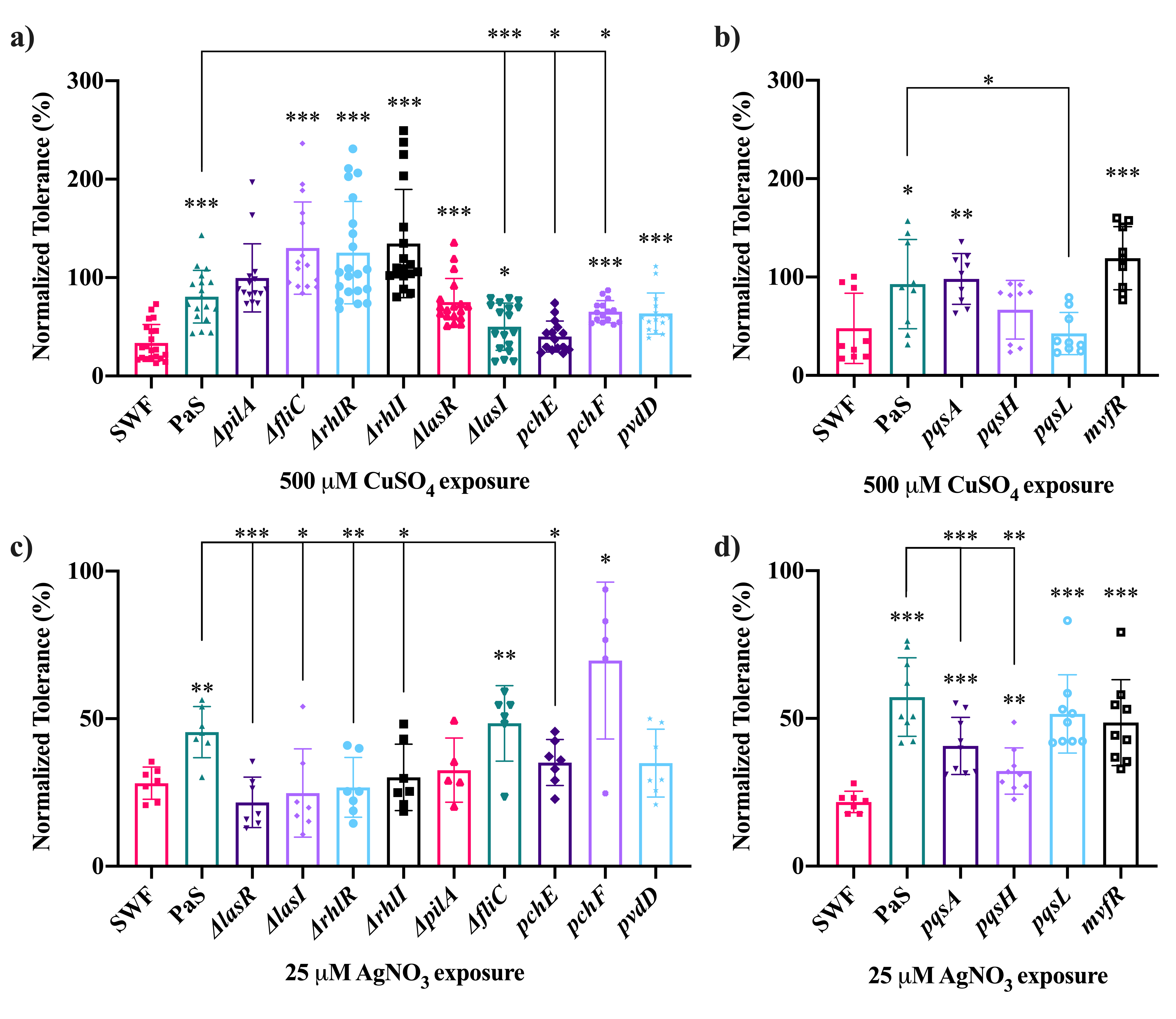

Supplement: FIG S8 [file mSystems.00746-20-sf008.tif]
