# Supplementary material for: Multiple Compounds Secreted by Pseudomonas aeruginosa Increase the Tolerance of Staphylococcus aureus to the Antimicrobial Metals Copper and Silver
Source: mSystems. 2020 Sep 8;5(5):e00746-20. doi: 10.1128/mSystems.00746-20 (PMC7483513; doi:10.1128/mSystems.00746-20)
Supplement: Supplementary file 2 [file mSystems.00746-20-st001.docx]

Metal Species		*S. aureus* alone	*S. aureus* with PaS
**Cationic Metals**	$AgNO_3$	16 µM	63 µM
	$CuSO_4$	500 µM	2000 µM
	$NiSO_4$	1000 µM	2000 µM
	$Ga(NO_3)_3$	>4000 µM	4000 µM
	$ZnSO_4$	500 µM	250 µM
	$CdSO_4$	500 µM	130 µM
	$AlSO_4$	4000 µM	4000 µM
**Anionic Metals**	K_2TeO_3	8000 µM	2000 µM
	Na_2SeO_3	>8000 µM	8000 µM
	$NaAsO_2$	2000 µM	250 µM
**Antibiotics and Antiseptics**	Nalidixic acid	200 µg/mL	50 µg/mL
	Tetracycline	6.25 µg/mL	6.25 µg/mL
	Benzalkonium chloride	12.5 µg/mL	1.5 µg/mL
	H_2O_2	1000 µM	130 µM
